# Influence of *SULT1A1* genetic variation on age at menopause, estrogen levels, and response to hormone therapy in recently postmenopausal white women

**DOI:** 10.1097/GME.0000000000000648

**Published:** 2016-08-05

**Authors:** Ann M. Moyer, Mariza de Andrade, Richard M. Weinshilboum, Virginia M. Miller

**Affiliations:** 1 Department of Laboratory Medicine and Pathology; 2 Division of Biomedical Statistics and Informatics; 3 Department of Molecular Pharmacology and Experimental Therapeutics; 4 Departments of Surgery and Physiology and Biomedical Engineering, Mayo Clinic, Rochester, MN.

**Keywords:** 17β-estradiol, Conjugated equine estrogens, Kronos Early Estrogen Prevention Study

## Abstract

**Objective:**

Onset and symptoms of menopause, and response to hormone therapy (HT) show large interindividual variability. *SULT1A1* encodes for a highly expressed enzyme that metabolizes estrogens. We evaluated the relationship between genetic variation in *SULT1A1*, menopause age, symptoms, and response to HT.

**Methods:**

Women enrolled in the Kronos Early Estrogen Prevention Study at Mayo Clinic were randomized to 48 months of treatment with oral conjugated equine estrogen (n = 34), transdermal 17β-estradiol (E_2_) (n = 33), or placebo (n = 35). Linear regression models and ANOVA were used to test for association of *SULT1A1* copy number, rs3760091, rs750155, and rs9282861 (*SULT1A1*^*∗*^*2*), with age at menopause and symptoms, levels of estrogens (estrone [E_1_], estrone sulfate [E_1_S], E_2_, and estradiol sulfate [E_2_S]), before and after HT.

**Results:**

*SULT1A1* gene copy number affected the minor allele frequency for each single nucleotide polymorphisms tested. Before administration of exogenous hormones, increasing number of G alleles at rs9282861 was associated with earlier age at menopause (*P* = 0.014), lower frequency of night sweats (*P* = 0.009), and less severe insomnia (*P* = 0.046). After 48 months of treatment, *SULT1A1* genotype was not associated with the presence of menopausal symptoms. In women randomized to oral conjugated equine estrogen, increasing number of the A allele at rs750155 was associated with lower E_1_S and E_2_S (*P* = 0.004 and 0.017), whereas increasing number of the C allele at rs3760091 was associated with lower E_2_S/E_2_ (*P* = 0.044).

**Conclusions:**

Interindividual variability in onset of menopause and symptoms before initiation of HT is explained in part by genetic variation in *SULT1A1* and may represent a step toward individualizing HT treatment decisions.

The onset of menopause, symptom severity, and the response to menopausal hormone therapy (HT) vary among women. This variation is thought to be multifactorial involving gene-environment interactions, with the heritability estimates of age at menopause ranging from 31% to 78%.^1-3^ Although menopause is an important health issue that will ultimately affect all women, the influence of genetic variation on menopausal physiology is currently not well understood.

As estrogen production by the ovaries decreases, circulating estrogen levels drop by approximately 90% and the conversion of androgens to estrogens by aromatase in the adipose tissue and skin becomes the predominant source of endogenous estrogens. Estrone (E_1_) and 17β-estradiol (E_2_) are both ligands for estrogen receptors (ERs), but ERs have higher affinity for E_2_, which is considered the main ligand for ER-mediated processes.^4^ Although estrogen conjugation is an important step in inactivation, conjugated estrogens may also have biologic activity and be important in delivery of estrogens to target tissues, where the sulfate moiety can be removed by sulfatase.^4,5^

Different formulations, doses, and routes of administration of HT may impact efficacy, with oral products being subjected to first-pass enterohepatic metabolism, which includes sulfation among other conjugation reactions.^4^ For optimal HT efficacy in symptom reduction and cardioprotection, as well as for avoidance of risks including cancers and thrombosis, maintaining the proper balance between estrogens and their conjugated forms may be important. It is unclear how genetic variation may affect serum concentrations of estrogens, symptom relief, or side effects in the setting of administration of these exogenous hormone products, which result in estrogen levels above physiologic menopausal levels.

At physiologic concentrations, E_1_ and E_2_ are substrates for SULT1A1, SULT1E1, and SULT2A1.^6^ Through the activity of these enzymes, estrone sulfate (E_1_S) is the form of endogenous estrogen circulating at the highest concentration.^7^ Although SULT1E1 has the highest affinity for estrogen substrates, the expression of SULT1A1 is ubiquitous, and it is the predominant sulfotransferase present in the liver, making it an important contributor to estrogen sulfation.^8^ At concentrations above physiologic concentrations, such as those seen in HT, the relative contributions of these enzymes to estrogen conjugation may differ. *SULT1A1* is highly polymorphic, with both a common copy number variation (CNV) and multiple single nucleotide polymorphisms (SNPs) (Fig. 1). SNPs and number of gene copies have both previously been shown to be associated with enzyme activity.^9,10^ Because SULT1A1 can metabolize estrogens, is highly expressed, and is genetically polymorphic, it is an ideal candidate gene for further study as a potential contributor to variability in menopausal physiology, as well as to the variability in response and side effects that result from HT.

**FIG. 1:**
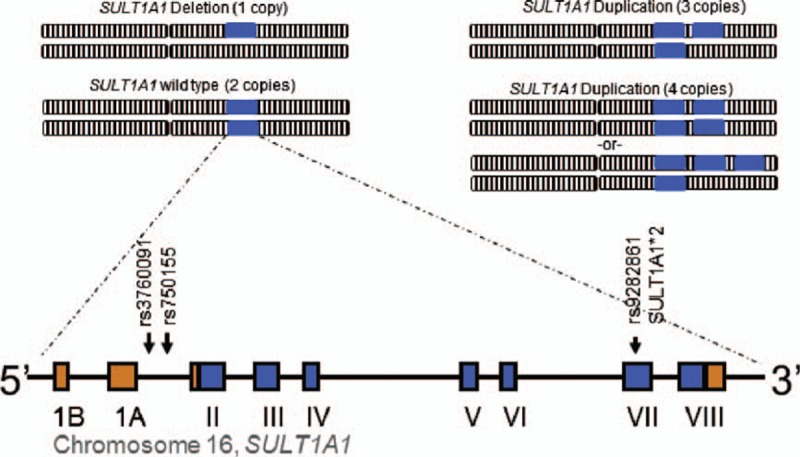
*SULT1A1* locus. *SULT1A1* is highly polymorphic with both a copy number variation and multiple single nucleotide polymorphisms. Copy number variation is demonstrated by each blue box representing a copy of the *SULT1A1* gene located on a chromosome (not to scale). Below, the location of the SNPs (arrows) are shown on a diagram of the gene, with coding exons depicted by the blue boxes, noncoding exons depicted by orange boxes, and introns and flanking regions shown as a line.

We hypothesized that genetic variation in *SULT1A1* may impact estrogen-related pathways that could contribute to individual differences in menopausal physiology and response to HT. Therefore, we analyzed the impact of *SULT1A1* genetic variation (CNV and SNPs) on onset of menopause and symptoms, as well as the levels of circulating E_1_, E_2_ and their sulfate conjugates in a subset of women enrolled in the Kronos Early Estrogen Prevention Study (KEEPS).^11^

## METHODS

### Participants

The participants included a subset of women enrolled in KEEPS at Mayo Clinic, Rochester, MN. On the basis of ancestry informative markers from a prior genotyping study, all of these women were of European ancestry (white).^12^ At enrollment, women averaged 52.7 (range 42-58) years of age and were 1.4 (0.5-3.0) years past menopause. Women were excluded if they had a hysterectomy, low-density lipoprotein cholesterol more than 190 mg/dL, body mass index (BMI) more than 35 kg/m^2^, preexisting coronary artery calcification (score >50 Agatston units), fasting glucose more than 126 mg/dL, uncontrolled hypertension (systolic blood pressure >150 mm Hg or diastolic blood pressure >95 mm Hg), or smoked more than 10 cigarettes/d. The participants were randomized to 48 months on one of three treatments: oral conjugated equine estrogen (oCEE; 0.425 mg/d), transdermal 17β-estradiol (tE_2_; 50 μg/d), both with micronized progesterone (200 mg/d for the first 12 d of the month) or placebo pills and patch.^13^ Menopausal symptoms, including night sweats, hot flashes, insomnia, and palpitations, were scored at baseline before administration of exogenous hormones and at the end of the 48-month treatment period and recorded on a scale of 0 to 5 (with 0 being no symptom and 5 being severe). Baseline clinical characteristics of the participants are shown in Table 1. Demographic and phenotypic characteristics of KEEPS participants did not differ across treatment assignments, and complete data are reported elsewhere.^13^ All participants provided written, informed consent, and the study was approved by the Mayo Clinic Institutional Review Board.

### Genotyping

*SULT1A1* genotype and copy number were determined as described previously on DNA extracted from blood drawn at baseline.^9^ Briefly, multiplex polymerase chain reaction was performed with primers that co-amplified a 212-bp fragment within *SULT1A1* and a 208-bp fragment within *SULT1A2*, followed by separation on an ABI 3730 (Thermo Fisher Scientific, Waltham, MA). The quantity of each fragment, represented by peak height, was measured using GeneMarker, version 1.51 (SoftGenetics, State College, PA), and the ratio between the peak heights was used to determine copy number.

The promoter SNPs, −624 (rs3760091) and −396 (rs750155), and ^∗^2 (Arg213His, rs9282861) were genotyped by pyrosequencing. The number of alleles with wild type (WT) and the variant nucleotide at each of the three loci was determined by combining copy number with the pyrosequencing peak height data. The allele ratio obtained from pyrosequencing was then plotted as “% signal” by copy number group to determine the quantity of each allele present.^14^

Previous reports of minor allele frequency (MAF) for each of the three SNPs studied did not take into account the gene copy number. Therefore, if the duplicated or deleted alleles were either WT or variant more frequently than identified in the samples with two gene copies, the reported MAF may be skewed from its true value. Therefore, MAF was calculated both with and without consideration of gene copy number in these samples.

### Measurement of serum steroids

All samples were collected from venous blood obtained in the morning after an overnight fast. These samples were not standardized as to time of taking/application of study treatments. A liquid chromatography-tandem mass spectrometry (LC-MS/MS) (Agilent Technologies, Inc, Santa Clara, CA) method was used to measure serum E_1_, E_1_S, E_2_ and estradiol sulfate (E_2_S) from serum samples from 102 participants drawn at the conclusion of the 48-month treatment period. The LC-MS/MS method was utilized because it uses an organic extraction to remove water-soluble conjugates, allows for concentration of the specimen, is free from interference, and represents a reference methodology.^15^

E_2_ and E_1_ were extracted from 0.5 mL of serum with the organic solvent methylene chloride. Deuterated E_2_-d5 and E_1_-d4 were added to each sample before the liquid extraction to serve as internal standards. After derivatization with dansyl chloride, high-pressure liquid chromatography was used before introduction of the derivatized sample extract into the tandem mass spectrometer.

E_1_S and E_2_S were extracted from serum via protein precipitation with acetonitrile. Deuterated E_1_S, d4-E_1_S, and E_2_S, d4-E_2_S, were added and used as internal standards. Positive pressure was used to push the sample into a 96 well collection plate, where water was added to each well. Analytes and internal standards were monitored on electrospray ionization mode and detected in multiplereaction monitoring mode. Intra- and interassay coefficients of variation were determined for dose ranges of 0.30 to 389 pg/mL for E_1_, 0.23 to 405 pg/mL for E_2_, 110 to 8,306 pg/mL for E_1_S, and 107 to 8,083 pg/mL for E_2_S. These coefficients of variation ranged from 9.9% (average across assays) at low concentrations to 2.9% (average across assays) at high concentrations. The limits of detection (in pg/mL) were 0.27 for E_1_, 0.04 for E_2_, 4.35 for E_1_S, and 4.31 for E_2_S.

### Statistical analysis

A multistep approach was taken to the analysis of this single candidate gene study. At baseline and after treatment, each symptom was reported on a scale of 0 to 5, with 5 being the most severe, but for our analyses, each symptom was analyzed as mild, defined as 0 to 2, or severe, defined as 3 to 5. Symptoms were dichotomized into mild or severe based on the impact to participants, with severe symptoms considered intolerable by most women. *SULT1A1* is polymorphic with both CNV and SNPs, which were combined for analysis. Because women had one to four copies of *SULT1A1*, the number of each nucleotide at each locus could range from 0 to 4. The nucleotide at each position previously reported to have higher activity in white individuals was selected (C at rs3760091, A at rs750155, and G at rs9282861), and the number of this nucleotide was used in our analyses.^10,16,17^ The estrogen levels and ratios of sulfate-conjugated to nonsulfated estrogens were log-transformed before analysis if they did not follow a normal distribution. All analyses were performed using R software version 3.0.1 or JMP version 10.^18,19^ Owing to both the stepwise approach taken and the exploratory nature of this candidate gene study, correction for multiple comparisons was not performed to minimize the chances of false negatives.

## RESULTS

### Genotyping

*SULT1A1* copy number was determined in 155 individuals screened for KEEPS. Eight participants demonstrated a single copy of the gene as a result of gene deletion (5.2%), 101 individuals had two copies of the gene (65.2%), 37 had three copies of the gene as a result of a duplication (23.9%), and 9 individuals had four copies (5.8%). None of the women had deletion of both copies of *SULT1A1*. The copy number frequencies in this population of white postmenopausal women are similar to previous reports of copy number frequency in white populations.^9^

The MAF for rs3760091, accounting for copy number, was 0.538, whereas it was 0.492 not accounting for copy number. Similarly, for rs750155 the MAF accounting for copy number was 0.554, and 0.479 not accounting for copy number. For ^∗^2 (rs9282861), the MAF was 0.286 and 0.243, accounting for copy number or not, respectively. The higher MAF for each variant when accounting for copy number suggests that many of the duplicated alleles contain variant nucleotides.

### Influence of *SULT1A1* on onset of menopause and symptoms

**FIG. 2:**
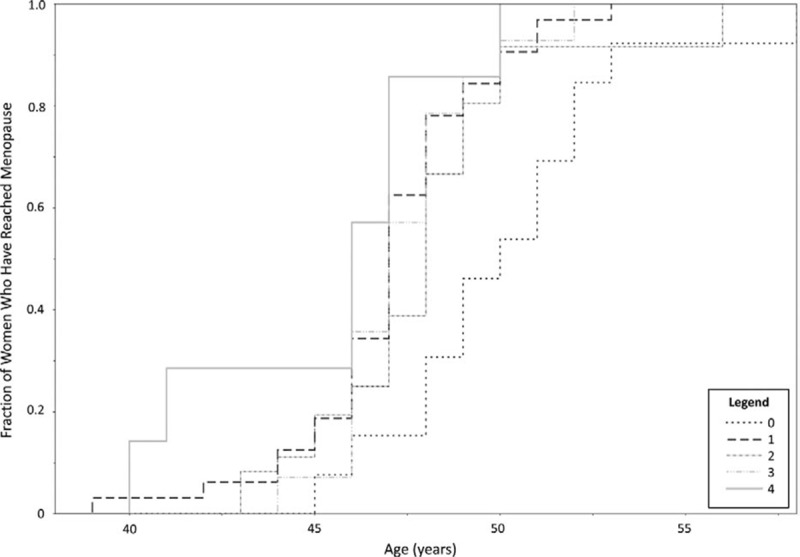
Age at menopause by number of G alleles at *SULT1A1* rs9282861. The fraction of women who have reached menopause is on the *y* axis, whereas age is on the *x* axis. Women are grouped by number of G alleles according to the legend. As the number of G alleles increased, women reached menopause at an earlier age.

Data were available for age at menopause in 102 women and symptoms in 100 women before randomization to treatment (baseline). Increasing number of G alleles at rs9282861, which would be expected to result in increased SULT1A1 activity, was associated with earlier age at menopause (ranging from an average age of 50.15 y among women with 0 G alleles to 45.29 y among those with 4 G alleles; *P* = 0.014), decreased frequency of night sweats (*P* = 0.009), and less severe insomnia (*P* = 0.046) **(**Fig. 2 and Table 2**)**. Interestingly, having 0 G alleles at this locus did not follow the trend for night sweats and instead women with no G alleles also showed lower frequency of night sweats. BMI or a diagnosis of metabolic syndrome may be confounding factors influencing age at menopause. In these participants, there was no association between *SULT1A1* copy number and BMI, nor between BMI and age at menopause (all *P* > 0.05). There was also no relationship between metabolic syndrome and *SULT1A1* copy number, nor between metabolic syndrome and age at menopause (all *P* > 0.05).

### Association of menopausal symptoms with *SULT1A1* genotype after HT

Before treatment (baseline), the symptom score reported by each woman did not differ by treatment group assignment (*P* = 0.66). Forty-eight months after randomization, menopausal symptom data were available for 75 women. There were no systematic differences between women with complete versus incomplete data in terms of baseline characteristics or treatment group assignment. At 48 months, menopausal symptom score (night sweats, hot flashes, insomnia, and palpitations) in the severe category and symptoms did not differ by treatment modality.

*SULT1A1* genotype was not significantly associated with the total number of symptoms after 48 weeks of treatment. Lower number of A alleles at rs750155, which would be expected to result in decreased SULT1A1 activity in whites, was associated with worsening insomnia from baseline to 48 months (*P* = 0.034). When each symptom was evaluated separately, women on HT were not experiencing any severe symptom, whereas two women in the placebo group continued to experience severe symptoms. Of these two women in the placebo group, one experiencing both severe hot flashes and insomnia had two copies of *SULT1A1*, and was heterozygous at rs3760091, homozygous A at rs750155, and homozygous G at rs9282861. The second woman was also experiencing insomnia and had three copies of *SULT1A1*, was homozygous (for all three copies) G at rs3760091, had 2 G and 1 A at rs750155, and had 2 G and 1 A at rs9282861.

### Effect of treatment modality on steroid hormone levels

Of the women who qualified for KEEPS and for whom serum was available for measurement of steroid hormones (n = 102), 34 were randomized to oCEE, 33 to tE_2_, and 35 to placebo pills and patch. The levels of E_1_, E_2_, E_1_S, and E_2_S were significantly different among the treatment groups, as were the ratios of E_1_S/E_1_ and E_2_S/E_2_ (Table 3). The oCEE group had the highest ratios of E_1_S/E_1_ and E_2_S/E_2_, whereas the tE_2_ group had the lowest E_2_S/E_2_ ratio. Treatment modality explains only 16% of the variation in E_1_S/E_1_ and 23% of E_2_S/E_2_, indicating that other variables, perhaps genetic variation, account for interindividual variation in levels of steroid hormones in these women.

### Association of steroid hormone levels with *SULT1A1* genotype

At the time of enrollment, only E_2_ level was available, which was not associated with age at menopause (*P* = 0.74), nor with night sweat or insomnia score (*P* = 0.87 and 0.46, respectively). Relationships between genetic variation in *SULT1A1* and levels of estrogens in the setting of administration of exogenous hormones in HT are shown in Table 4. Significant effects of *SULT1A1* genotype on hormone levels were only observed in the oCEE group. With increasing number of the C allele at rs3760091, the ratio of E_2_S/E_2_ was lower (*P* = 0.044) in the women taking oCEE. With increasing number of the A allele at rs750155, there were decreasing levels of all estrogens and estrogen sulfates. Women with 0 or 1 A had higher levels and those with 2 and 3 A alleles had lower levels, but this was only significant for E_1_S (*P* = 0.004) and E_2_S (*P* = 0.017). Only one individual in this treatment group had 4 A alleles at this locus, making it difficult to assess the impact of this genotype. Despite the finding that the number of G alleles at rs9282861 was associated with age at menopause and symptoms before HT, after 48 months of HT or placebo, no association was identified between this locus and estrogen levels in any of the treatment groups.

## DISCUSSION

Using a candidate gene approach, we identified an association between the number of *SULT1A1* G alleles at rs9282861 and age at menopause, as well as insomnia and night sweats before initiation of HT. In addition, we identified a relationship between *SULT1A1* genetic variation and levels of estrogens and the ratio of sulfate-conjugated estrogens to nonconjugated estrogens among women taking oCEE HT.

The genetic influence on menopausal symptoms is complex and multifactorial, with many genes and pathways interacting with each other and the environment. Owing to its role in estrogen metabolism, we took a candidate gene approach to study the influence of variation in *SULT1A1* on menopausal timing and symptoms, and hormone levels. SULT1A1 activity varies among individuals and is in part responsible for metabolizing estrogens. In addition to sulfation increasing the water solubility of estrogens for excretion,^20^ sulfated estrogens have a longer half-life than the unconjugated form and are considered to be an inactive “storage pool” in equilibrium with nonsulfated estrogens.^4,5^ Although in isolation, increased SULT1A1 activity would be expected to result in decreased bioavailability of estrogens, biological systems and estrogen pathways are complex with many additional variables contributing to variation in estrogen levels. In addition, it is unknown at this time how genetic variations in pathways regulating estrogen metabolism modulate and interact with genetic variations of other pathways that are involved in the phenotypic expressions of symptoms, including those involved with production and degradation of neurotransmitters, norepinephrine and serotonin, and their respective receptors.

In addition to CNVs, *SULT1A1* contains three SNPs that are commonly studied and may affect hormonal efficacy. Two SNPs, rs3760091 (−624) and rs750155 (−396), are located in the promoter region and are in linkage disequilibrium with the *SULT1A1*^*∗*^*2* (Arg213His, rs9282861) polymorphism.^16,21^ The C nucleotide at rs3760091 creates a putative Sp1 binding site and is found in haplotypes that are associated with increased SULT1A1 activity in platelets.^16^ The rs750155 promoter SNP shows ethnic variation in its association with biologic effect—the A nucleotide is associated with decreased platelet SULT1A1 activity in African-Americans and with increased platelet SULT1A1 activity in white populations of European ancestry.^16^ The *SULT1A1*^*∗*^*2* polymorphism (G > A single nucleotide change) is associated with decreased enzyme activity and decreased thermal stability of the enzyme.^10,17^ In an individual, gene CNV and SNPs occur together and cannot be separated, so analyzing the effect of allele “dosage” may be more biologically relevant than separately analyzing CNV and SNPs. Here, we found a different MAF for each of the three SNPs studied when the copy number was taken into account compared with only analyzing each locus as homozygous WT, heterozygous, or homozygous variant. These results highlight the importance of investigating both types of variation.

In this study, increasing number of G alleles at rs9282861 was associated with earlier age at menopause, as well as less severe insomnia and decreased frequency of night sweats before initiation of HT. Although we hypothesize that these findings are related to changes in estrogen levels as a result of this genotype, we were unable to test this hypothesis because at baseline only E_2_ was measured, and women were menopausal for at least 6 months before enrollment in this study. SULT1A1 has many substrates in addition to estrogens, so it is also possible that the observed relationships are due to the impact of SULT1A1 variation on other substrates. For example, a recent study demonstrated that SULT1A1 sulfates melatonin, which could impact postmenopausal insomnia in addition to estrogen.^22^ Further study of these associations may clarify the underlying mechanisms responsible. There was approximately a 5-year difference in mean age at menopause among women with 0 G alleles compared with women with 4 G alleles at rs9282861. The impact of age at menopause on risk for a variety of disorders, including breast cancer and depression, has been widely studied. In addition, younger age at menopause is associated with increased risk of cardiovascular disease.^23^ Therefore, our finding of an association between *SULT1A1* genotype and age at menopause warrants further study in other cohorts and especially with regard to other processes associated with aging.

Although we hypothesized that *SULT1A1* genetic variation would be associated with hormone levels after HT, and thus with clinical outcomes, and we did identify several associations between genotype and hormone level and between genotype and clinical symptoms, we did not observe a clear relationship linking all three together in the presence of HT. This may be due to our small sample size, small or no effects of this gene, and/or the multifactorial nature of these phenotypes. In addition to estrogen metabolism that we have studied, prior studies have investigated the impact of genetic variation in ERs,^23-28^ and a recent genome-wide association study identified SNPs in a transporter to be highly correlated with both the level of conjugated estrogens and the ratio of conjugated to unconjugated estrogens.^29^

Although several relationships between *SULT1A1* genotype and menopausal timing and symptoms were identified, our study had several limitations. First, our sample size was small and women were randomized to one of three treatments further decreasing the power to detect genotypic effects on the phenotypes of interest. Second, although the collection of samples for hormone measurement was standardized, the timing with which women took pills or applied a patch was not controlled and could contribute to variability in estrogen levels. Finally, in our study, the participants were a relatively homogenous population of healthy white women of European ancestry. The impact of *SULT1A1* genetic variation on hormone levels and menopausal symptoms may vary by ethnic background, limiting the generalizability of our findings to non-white populations, and requires further study in other populations.

## CONCLUSIONS

Understanding the molecular mechanisms underlying variation in menopausal timing and symptoms is crucial to improving many aspects of women's health, including risk of cancer and cardiovascular disease. With recent advances in understanding the human genome, it is now possible to begin to understand the impact of genetic variation on menopause, which may translate into models to predict which women may gain the most benefit from HT and to optimize the dose and formulation, as well as to predict which women may experience harmful side effects. Although there is a vast chasm between current knowledge and the goal of optimizing HT to improve women's health, this study represents an initial step toward the understanding of the impact of genetic variation on estrogen metabolism and the individualization of HT.

## Figures and Tables

**TABLE 1:** Baseline characteristics of participants

Age at enrollment, y	52.4 (0.2); 45-58
Age at menopause, y	47.7 (0.3); 39-58
Body mass index, kg/m^2^	27.0 (0.4); 18.1-35.9
Waist circumference, cm	84.2 (1.2); 63-118
Fasting blood glucose, mg/dL	92.2 (0.8); 71-112
Total cholesterol, mg/dL	216.3 (3.1); 132.0-293.0
HDL-cholesterol, mg/dL	61.8 (1.6); 38-112
LDL-cholesterol, mg/dL	131.1 (3.0); 58.0-190.0
Triglycerides, mg/dL	92.7 (4.4); 27-270
Systolic BP, mm Hg	121.7 (1.4); 88-152
Diastolic BP, mm Hg	75.4 (0.8); 60-93

Data are presented as mean (standard error) and range. BP, blood pressure; HDL-cholesterol, high-density lipoprotein cholesterol; LDL-cholesterol, low-density lipoprotein cholesterol.

**TABLE 2:** *P* values for association of *SULT1A1* genotype with age at menopause and baseline postmenopausal symptom scores

Characteristic	No. of C at rs3760091	No. of A at rs750155	No. of G at rs9282861
Age at menopause	0.82	0.57	**0.014**
Severity of night sweats, hot flashes, insomnia, and palpitations combined	0.83	0.83	0.11
Night sweats	0.13	0.07	**0.009**
Hot flashes	0.55	0.35	0.47
Insomnia	0.40	0.60	**0.046**
Palpitations^*a*^	N/A	N/A	N/A

Statistically significant associations (*P* < 0.05) are in bold.

^*a*^All women were in the “low” group for palpitation symptom score.

**TABLE 3:** Estrogen levels and ratios by treatment group

Hormone	oCEE	tE_2_	Placebo	*P*
E_1_, pg/mL	61.4 (4.1); 9.6-153.0	35.5 (4.2); 9.9-72.0	19.6 (4.0); 9.0-56.0	<0.01
E_1_S, pg/mL	2160.1 (202.6); 138.0-7718.0	992.7 (212.2)^*a*^; 91.0-3226.0	347.6 (199.7); 23.0-1408.0	<0.01
E_1_S/E_1_	34.0 (2.8); 7.7-111.9	24.3 (2.9)^*a*^; 4.1-56.6	16.1 (2.8); 2.6-40.3	<0.01
E_2_, pg/mL	12.1 (2.8); 2.9-25.0	32.3 (2.8); 3.2-131.0	5.8 (2.7); 3.0-12.0	<0.01
E_2_S, pg/mL	25.9 (3.0); 4.9-84.9	24.3 (3.1)^*a*^; 4.9-110.9	7.6 (2.9); 4.9-19.0	<0.01
E_2_S/E_2_	2.1 (0.17); 0.5-7.2	1.0 (0.18)^*a*^; 0.1-3.7	1.3 (0.17); 0.6-2.7	<0.01

Data are shown as mean (standard error) and range; *P* value for the difference among groups for each analyte. E_1_, estrone; E_1_S, estrone sulfate; E_2_, 17β-estradiol; E_2_S, estradiol sulfate; oCEE, oral conjugated equine estrogen; t, transdermal.

^*a*^2 missing values.

**TABLE 4:** *P* values for association of each hormone or ratio with number of high activity allele for each SNP, by treatment group

	No. of C at rs3760091			No. of A at rs750155			No. of G at rs9282861		
Hormone	oCEE	tE_2_	Placebo	oCEE	tE_2_	Placebo	oCEE	tE_2_	Placebo
E_1_	0.43	0.32	0.65	0.09	0.77	0.44	0.19	0.97	0.41
E_1_S	0.26	0.56	0.43	**0.004**	0.28	0.33	0.13	0.61	0.81
E_1_S/E_1_	0.24	0.75	0.74	0.17	0.15	0.21	0.47	0.42	0.74
E_2_	0.73	0.45	0.63	0.28	0.46	0.90	0.31	0.79	0.62
E_2_S	0.18	0.53	0.53	**0.017**	0.27	0.23	0.29	0.16	0.42
E_2_S/E_2_	**0.044**	0.54	0.21	0.33	0.44	0.06	0.86	0.97	0.54

Statistically significant associations (<0.05) are in bold. E_1_, estrone; E_1_S, estrone sulfate; E_2_, 17β-estradiol; E_2_S, estradiol sulfate; oCEE, oral conjugated equine estrogen; SNP, single nucleotide polymorphism; t, transdermal.
